# Systematic biology analysis on photosynthetic carbon metabolism of maize leaf following sudden heat shock under elevated CO_2_

**DOI:** 10.1038/s41598-018-26283-x

**Published:** 2018-05-18

**Authors:** Mingnan Qu, Genyun Chen, James A. Bunce, Xiaocen Zhu, Richard C. Sicher

**Affiliations:** 1 CAS Center for Excellence in Molecular Plant Sciences, Institute of Plant Physiology and Ecology, Shanghai Institutes for Biological Sciences, Chinese academy of Sciences, 200032 Shanghai, China; 2 USDA-ARS, Crop Systems and Global Change Laboratory, Beltsville, MD 20705 USA; 3 Human Phenome Institute, Fudan University, Shanghai, 200438 China

## Abstract

Plants would experience more complex environments, such as sudden heat shock (SHS) stress combined with elevated CO_2_ in the future, and might adapt to this stressful condition by optimizing photosynthetic carbon metabolism (PCM). It is interesting to understand whether this acclimation process would be altered in different genotypes of maize under elevated CO_2_, and which metabolites represent key indicators reflecting the photosynthetic rates (P_N_) following SHS. Although B76 had greater reduction in P_N_ during SHS treatment, our results indicated that P_N_ in genotype B76, displayed faster recovery after SHS treatment under elevated CO_2_ than in genotype B106. Furthermore, we employed a stepwise feature extraction approach by partial linear regression model. Our findings demonstrated that 9 key metabolites over the total (35 metabolites) can largely explain the variance of P_N_ during recovery from SHS across two maize genotypes and two CO_2_ grown conditions. Of these key metabolites, malate, valine, isoleucine, glucose and starch are positively correlated with recovery pattern of P_N_. Malate metabolites responses to SHS were further discussed by incorporating with the activities and gene expression of three C_4_ photosynthesis-related key enzymes. We highlighted the importance of malate metabolism during photosynthesis recovery from short-term SHS, and data integration analysis to better comprehend the regulatory framework of PCM in response to abiotic stress.

## Introduction

Increasing atmospheric CO_2_ concentration has led to higher surface temperatures^1^. Hereby, two different types of temperature stress are known: an increase in the mean temperature and an increase in the frequency of sudden heat shock (SHS)^2^. This SHS can also trigger dramatically detrimental effects on crop growth and final yield^3^. The reduction of final yield in crops depends on the responsiveness of plants not only during, but also after SHS. Photosynthetic efficiency (P_N_) is an essential indicator reflecting growth status in response to heat stress, and previous studies have shown that the recovery of P_N_ in plants from SHS is related to ribulose-1,5-bisphosphate carboxylase/oxygenase (Rubisco) activase, Oxygen evolving complex (OEC), protein contents of stroma and the thylakoid membrane and the photochemical efficiency of photosystem II^4–7^. However, the knowledge about plants response to SHS is still limited and controversial.

Metabolite analysis is an effective and quantitative method to elucidate the mechanisms of abiotic stress tolerance, including heat stress^8^. Heat stress can induce the changes of various metabolites such as organic acids, amino acids and carbohydrates, which have important functions involved in photosynthesis and respiration^9^. These compounds are involved in various metabolic functions within the plant such as regulating plant-water relations, signaling pathways, and the protein synthesis as well as defense systems against stress^10^. A reduction in the levels of total non-structural carbohydrates under heat stress has been reported in different plant species, such as sucrose and starch in winter wheat (*Triticum aevistum* L.), Indian mustard (*Brassica juncea* L.), crested wheat grass (*Agropyron cristatum* L., Gaertn.) and redtop (*Agrostis alba* L.)^11–13^. In terms of organic acids and amino acids, it has been reported that the abundance of many metabolites, such as γ-aminobutyric acid (GABA), β-alanine, alanine, and proline in cowpea (*Vigna unguiculata*) as well as malic acid, citric acid and threonic acid in Kentucky bluegrass (*Poa pratensis*) can be enhanced by heat stress^14–16^. However, the studies on reprogramming metabolism after SHS is still less reported, and the accumulation or depletion of metabolites after SHS could also affect recovered capacity of photosynthetic efficiency.

Recently, an important question about whether elevated CO_2_ can mitigate heat tolerance raises extensive debate and remains controversially. In a few C_3_ species, elevated CO_2_ has been shown to enhance photosynthetic tolerance to high temperatures through improving photochemical efficiency, resource allocation to light harvesting, and water use efficiency^17–19^. In some C_4_ species, previous reports have demonstrated that elevated CO_2_ has decreased the tolerance of the photosynthetic machinery to high temperatures^20^. These decreases in the photosynthetic efficiencies have generally been attributed to the diversification of the mechanisms of its response to heat stress; including stomatal limitations^18,20^, cellular membrane stability, photosynthetic efficiency^21^, and photosynthetic enzyme stability^4,22^. However, few studies have investigated the changes in metabolites accumulation in response to heat stress under elevated CO_2_ conditions, despite some studies that reported the positive effects of elevated CO_2_ on plant growth under detrimental environments such as drought stress and fertilizer deficiency^23,24^.

Accumulative evidences have suggested that there exists tremendous variation within and between species in coping with the heat stress^25^. In maize, field investigations have proven that B76 and B106 are two genotypes possessing distinct photosynthetic performance under heat stress^26^. In this study, we inquire whether two maize genotypes possess different response after SHS, and which primary metabolites can be used as indictors reflecting recovery pattern of P_N_ after SHS. Using GC-MS, we quantitatively determined various metabolites involved in TCA, glycolysis, photorespiration and amino acids metabolism following SHS treatments. To determine the key metabolites that can explain the response of P_N_ following SHS treatments, we applied a stepwise feature extraction approach via linear regression model. Finally, we summarized the key metabolic pathway regarding heat response in both maize genotypes studied herein. The representative framework of metabolic pathways will help us better understanding the specific response of plants in future climatic changes.

## Materials and Methods

Two maize (*Zea mays L*.) genotypes, B76, and B106 were used in this study, which were developed at Iowa State University to resist European corn borer^27^. We obtained seeds of B76 (PI 550483) and B106 (PI 594049) from U.S. Germplasm Resources Information Network (GRIN: http://www.ars-grin.gov/).

The two genotypes were grown in open top chambers (OTCs) in the field. The experimental site was located at USDA, South Farm of Beltsville Agricultural center, USDA-ARS (39° 00′ N, 76° 56′ W). Six OTCs were used and each OTC covered 1.2 m × 2.3 m of ground area with 2 m high. Each chamber was spaced from the nearest one by 2 m to avoid or at least minimize shading effect, and individual plants were thinned at 7 days after emergence (DAE) spaced by 15 cm distance between each other. Pre-mixed CO_2_ is blown through OTCs horizontally, and each OTC was equipped with four powerful fans to ensure homogeneous temperature and CO_2_ concentration. Soil was kept regularly moist to field capacity by watering once a week. Plants in OTCs were exposed to ambient CO_2_ (mean of 394 µmol mol^−1^) air or to ambient plus 180 µmol mol^−1^ CO_2_. There were three open top chambers (OTCs) for each CO_2_ treatment, and all chambers contained both maize cultivars (B76 and B106). Two planting dates (May 24^th^ and June 14^th^, 2013) were conducted in the field study to increase replications number. Mean daily air temperature for the first planting was 23.8 °C, and it was 25.0 °C for the second planting. Maximum temperatures of 37.6 and 37.9 °C occurred on June 29^th^ and July 30^th^, respectively. A sudden heat shock (SHS) treatment, described below, was applied when the sixth leaf was fully expanded at around 30 DAE.

### Heat stress treatments

**Figure 1:**
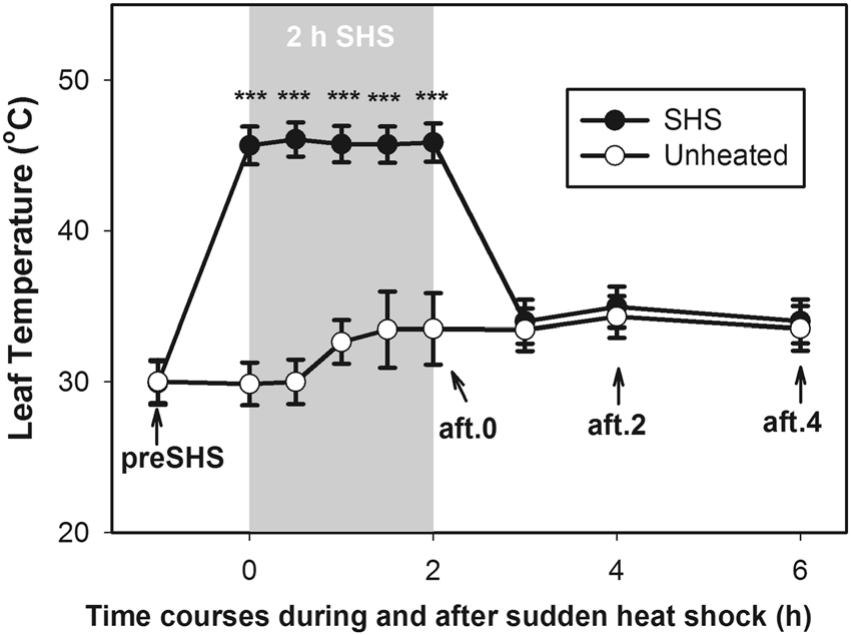
Time courses of leaf temperature during and after 2 h sudden heat shock (SHS) cycles across both CO_2_ treatments (380 μmol mol^−1^, 560 μmol mol^−1^). Grey area represents the period during 2 h SHS at 45–46°C. Arrows represent time points that gas exchange data and leaf sampling measured. In open top chambers, gas exchange measurements prior to SHS started from 9:30am–10:00 am, while SHS was from 10:00 am–12:00 pm. After SHS at 2 h and 4 h, the local time was 14:00 pm and 16:00 pm, respectively. Numbers of replications, n = 5–13. Pairwise t-test was used to compare significant difference of leaf temperature between SHS and nonheated treatments. Symbol “***” represents *P* value < 0.001.

A sudden heat shock (SHS) treatment of 2 h (10:00 am–12:00 pm) was applied on an intact leaf for each plant in the field for both maize genotypes. For each CO_2_ growth condition (ambient and elevated CO_2_), an intact undetached fully-expanded leaf from each maize cultivar was inserted in the same water jacketed cuvette at leaf temperature of around 46 °C. The microclimate inside the cuvette was maintained similar as macroclimate condition of the OTC during SHS treatments. Leaves were then removed out from the cuvettes for measurements after recovery periods of 0, 2 and 4 h. The averaged leaf temperature before placing leaves in the cuvettes was 26 °C. After 2 h SHS treatment, air temperatures increased to about 32 °C (Fig. 1). We exposed leaves to SHS for 2 h because this artificial procedure can mimic the waves time of high temperature occurring frequently in the field, and it allow us to examine recovery periods of 2 and 4 h within a single day. During each conducted experiment, two batches of sixth fully expanded leaves were exposed for 2 h to SHS, then one batch was used immediately for gas exchange measurements and from the other one, leaves were harvested for metabolites analysis, as described in detail below.

### Gas exchange measurements

Steady-state net photosynthetic rates (*P*_N_), stomatal conductances (*g*_s_), transpiration rates (*E*), and internal cellular CO_2_ concentration (*C*_i_) of leaves was measured with a portable infrared gas analyzer (CIRAS-1, PP Systems, Amesbury, MA). Measurements were made a few minutes before insertion of leaves into the cuvette (known as control), a few seconds after their removal from the cuvette (labeled as 0 h), and at 2 and 4 h following SHS application as evidently described above. Leaves were measured at the same CO_2_ levels at which the plants were grown (either 380) and at elevated CO_2_ of 560 µmol mol^−1^ CO_2_ (380 + 180). All measurements were conducted on sunny days, and sunlight photosynthetic photon flux densities (PPFD) was at least 1500 µmol m^−2^ s^−1^ for the gas exchange measurements.

### Metabolite measurements

Freezed-dried leaf tissue (~30 mg total) for each treatment was added to 2.0 mL Eppendorf tube containing 3.2 mm ceramic beads and 100 µl of fine garnet powder. Maize leaf tissue was homogenized in a Tissue Lyzer ball mill at 30 cycles s^−1^ as previously described^28^. A 50 µl mixture of 2.5 mM *α*-aminobutyric acid, 2.0 mg ribitol and 1.4 mL ice-cold 70% methanol were injected into each sample and vortexed altogether. The suspended plant tissue was heated to 45 °C for 15 min in a water bath. These extracts were centrifuged for 5 min at 12.000 *g* in an Eppendorf microcentrifuge. Supernatants were gently transferred into 15 mL fresh conical, plastic centrifugation tubes. The pellets were washed once with 70% methanol as described above and the supernatants were combined. After washing, the pellets were air-dried overnight and used for the determination of starch as previously described^29^.

Organic acids, amino acids and soluble carbohydrates were measured by gas chromatography coupled to mass spectrometry (GC-MS) as described elsewhere^30^. Derivatized samples were separated by gas chromatography and the resultant ions were detected with a mass selective detector (model 7125, Agilent technologies, Wilmington, DE). Total ion chromatograms were quantified using peak identification and calibration parameters within the Agilent MSD Chemstation software program. Independent standard curves were prepared for each set of extractions with known mixtures of organic acids, amino acids and soluble carbohydrates. Ribitol added during extraction functions as the internal standard. The compounds included in the organic acid fraction were; 2-oxoglutaric, quinic acid, adipic acid, shikimic acid, pyruvic acid, citric acid, aconitate, maleic acid, malic acid, oxalic acid, malonic acid, glyceric acid, fumaric acid and succinic acid. Compounds in the soluble carbohydrate fraction were ribose, fructose, glucoe, myo-inositol, sucrose, maltose, trehalose and raffinose and starch. The compounds present in the amino acids fraction were; leucine, Isoleucine, alanine, glycine, serine, valine, threonine, proline, putrescine, asparagines, glutamine and phenylalaine.

### Feature extraction approach

Relatedness among metabolites was analyzed by Pearson correlation coefficient. Furthermore, we applied feature extraction approach to unravel the key metabolites that can largely explain P_N_ variance in response to SHS and CO_2_ treatments in two maize genotypes. The model is as defined follows:

1 $$y={\beta }_{1}{x}_{1}+{\beta }_{2}{x}_{2}+\ldots {\beta }_{v}{x}_{v}+\varepsilon $$

where *y* is a vector representing biomass values of each rice accession, *x* is a vector of independent variables, *β* is weighted coefficients corresponding to *x*, and *ε* is an error vector. The model was constructed with a stepwise manner, which can identify highly relevant parameters and remove low relevant parameters based on the Akaike information criterion (AIC) as previously described^31^. In practice, a training dataset including 80% items of the whole dataset was randomly extracted from the original dataset and the remaining 20% data were used as a test dataset^31,32^. The training dataset was first defined to build the regression model, and then an independent validation was conducted on the test dataset to check performance of the model.

### Photosynthetic enzyme assays

In this study, to better understand C_4_ photosynthetic metabolism responded to SHS treatments, three key enzymes involving in C_4_ photosynthesis were determined, including PEP carboxylase (PEPCase), NADP-malic enzyme (NADP-ME) and NADP-malate dehydrogenase (MDH). Five leaf discs (about 3.14 cm^2^) were removed from the lamina of the leaves in the field experiments quickly after SHS. Leaf material was rapidly transferred to labeled envelopes and immediately immersed in liquid nitrogen to quench metabolism. All samples were stored for a maximum of 1 month at −80 °C prior to analysis. Two leaf discs from each plant were extracted with 0.6 mL ice cold extraction buffer consisting of 50 mM Tris–HCl (pH 7.50), 10 mM MgCl_2_, 1 mM EDTA, 1% (w/v) PVP-40, 5 mM Na^+^-pyruvate and 10% glycerol. Immediately prior to extraction, 1 μM leupeptin and 5 mM dithiothreitol were added to the solution. Two leaf discs were extracted at 0 °C with a ground glass tissue homogenizer and the homogenates were transferred to 2 mL plastic centrifuge tubes, and spun for 3 min at full line voltage in microfuge (340 rpm). The supernatant was transferred to a 1.5 mL Eppendorf tube and assayed immediately or stored in liquid nitrogen (N_2_).

Enzyme activity measurements were performed spectrophotometrically at 25 °C, and calculated from the rate of change in optical density at 340 nm. NADP-malate dehydrogenase (MDH) was measured in 1 mL solution containing 50 mM Tris-HCl (pH 8.0), 1 mM EDTA, 100 mM oxalacetic acid, 10 mM NADPH and 0.025 mL leaf extract^33,34^. PEP carboxylase (PEPCase) activity was measured in 1 mL solution containing 50 mM Tris–HCl (pH 8.0), 5 mM NaHCO_3_, 5 mM MgCl_2_, 0.14 mM NADH, 10 mM PEP (tricyclohexlamine salt), 1 U Malate dehydrogenase and 0.025 mL sample as reported previously^35^. NADP-malic enzyme (NADP-ME) was measured in 1 mL solution containing 50 mM Tris-HCl (pH 8.0), 5 mM EDTA, 500 mM MgCl_2_, 100 mM malic acid, 250 mM dithioerythritol, 20 mM NADP and 0.025 mL sample. All measurements were performed using a Shimadzu model 2101 spectrophotometer operated in the kinetic mode.

### Quantitative transcript measurements

To compare the activities of three C_4_ photosynthesis-related key enzymes, i.e, *PEPC*, *MDH* and *NADP-ME* as mentioned above, the expressions of all gene were also determined. Two maize leaf disc [approximately 0.5 g fresh weight (FW)] were ground using liquid nitrogen in a sterile mortar and pestle, and total RNA was extracted using TRIzol® reagent according to the manufacturer’s instructions (Invitrogen, Carlsbad, CA). RNA was quantified with a NanoDrop spectrophotometer (model 2000c, Thermo-Fisher Scientific Inc., Waltham, MA). First strand cDNA was synthesized with 2 μg of total RNA (OD260 nm/OD280 nm > 1.95), oligo (dT) 20 primers and SuperScript III RNase H reverse transcriptase from Invitrogen. The resultant cDNA was diluted 10-fold and was used as a template for real-time quantitative polymerase chain reaction (qPCR). Amplifications were performed with a model Mx3005P qPCR System plus Brilliant SYBR® Green QPCR Master Mix (Stratagene, La Jolla, CA). Details of the qPCR procedures were described previously^36^. Primers and functional annotations for three genes are listed in Table 1. The maize actin 1 gene was used as an expression control and sequences of its forward and revered primer are CTATGTTCCCTGGCATTGCT and GGGCCCAAAGAATTAGAAGC, respectively, as described previously^37^. Assays were performed with four biological samples from each treatment, and measurements were replicated three times.

**Table 1:** Primer information of three C_4_ key photosynthetic enzymes.

GenBank#	Name	Sequence	Product length (bp)
HQ697600.1	*NADP-ME-F*	AGGCTCTCTTCAGCCATTCA	173
	*NADP-ME-R*	TAGGCCTCTCGTTGAAGGAA	
JF810422.1	*NADP-MDH-F*	GGGAAGTCAGCATTGGCATAG	192
	*NADP-MDH-R*	CAACAACTAAGACTTTCGCGT	
HQ697599.1	*PEPC-F*	GAGATCCAAGCAGCCTTCAG	215
	*PEPC-R*	CCACCCATCCAAGAAGAGAA	

## Results

### Responses of gas exchange parameters to sudden heat shock under elevated CO_2_

**Figure 2:**
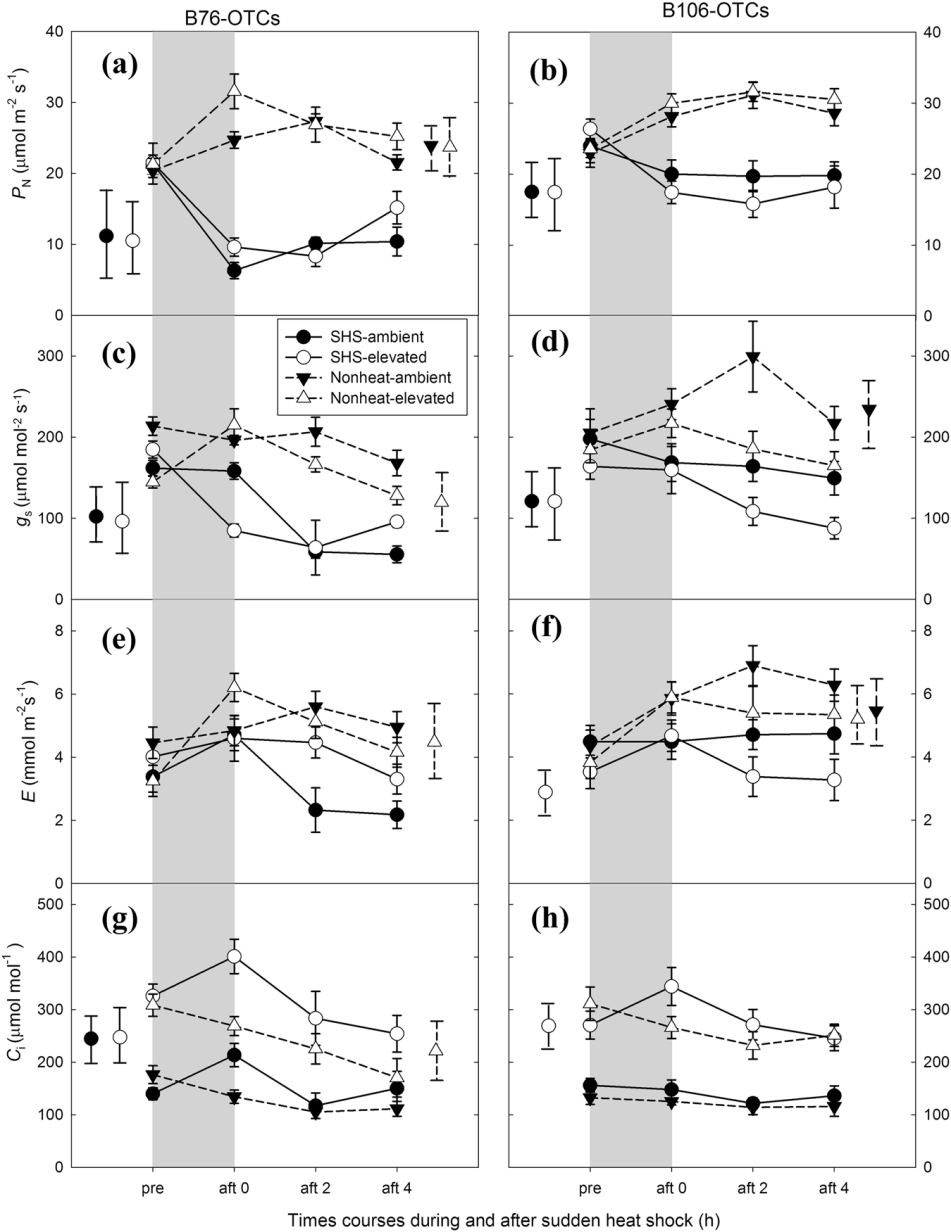
Sudden heat shock (SHS) induced decrease and recovery of photosynthetic variables in two maize genotypes grown under elevated CO_2_ conditions. Shapes of Shapes of circle in solid line and triangle in dotted line represent SHS treated leaves and nonheated leaves, respectively, while black and white symbols mean ambient CO_2_ and elevated CO_2_, respectively. The grey area represents the period during 2 h SHS. *Vertical bars* represent at two sides of each panel represent significance scale regarding each combination of CO_2_ and SHS treatments across during and after SHS from one-way *ANOVA* analysis, (*P* < 0.05). n = 5.

The *P*_N_ in B76 following a 2 h sudden heat shock (SHS) treatment decreased by averaged 62% across two CO_2_ growth conditions, the reduction of which was twice more than that observed in B106 (Fig. 2a,b). P_N_ value was around 20 μmol m^−2^s^−1^ in B76 under both CO_2_ condition before SHS, and decreased by 60% and 50% at ambient CO_2_ and elevated CO_2_, respectively, after removal of SHS at 0 h. In contrast, P_N_ in B106 reached around 26 μmol m^−2^s^−1^ for both CO_2_ condition before SHS, and decreased by 20% and 40% at ambient CO_2_ and elevated CO_2_, respectively, after removal of SHS at 0 h. The recovery of *P*_N_ from SHS in B76 was faster than B106 irrespective of CO_2_ effects. In terms of B76, the decrease in *P*_N_ caused by the SHS was similar or less at elevated than at ambient CO_2_ after SHS at 4 h compared with that at 0 h, while in B106, the greater reduction in P_N_ was observed at elevated than at ambient CO_2_ (Fig. 2a,b). Stomatal conductances (*g*_s_), transpiration rates (*E*) and vapor pressure deficit (VPD) decreased after immediate removal of SHS (0 h) across maize genotypes (Fig. 2a,b; Fig. S1). However, the reduction in *P*_N_ cannot be attributed to the effects of stomatal limitation because the internal CO_2_ concentration (Ci) after SHS at 0 h was above its initial value recorded before SHS. Interestingly, the lower values in *g*_s_ in heat stressed leaves of B76 and B106 remained similar even after withdrawing them from SHS by about 2 and 4 h.

### Response of metabolites after sudden heat shock under two grown CO_2_ conditions

**Figure 3:**
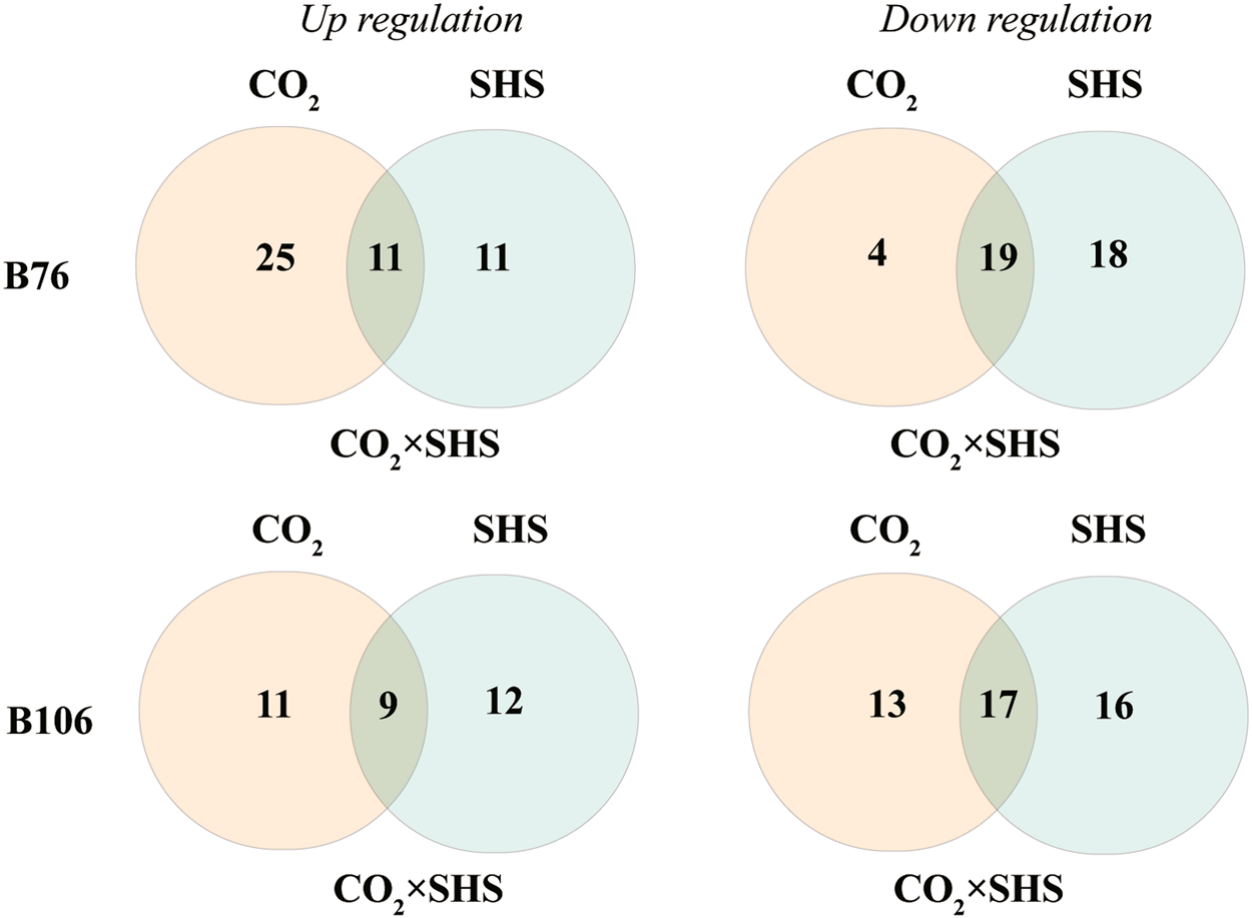
Venn diagram showing 35 metabolites in response to CO_2_ and sudden heat shock (SHS) at 0 h and their interactions in two maize genotypes. Overlapped areas represent metabolic response to interaction of SHS and CO_2_.

To further understand plant performance in response to SHS, 35 primary metabolites were determined. Metabolites in two maize genotypes exhibited differential responses to either a single treatment of elevated CO_2_ or SHS (at 0 h) or their combined effects (Fig. 3; Tables S1–S2). Results from Venn diagram (Fig. 3) indicated that 25 and 4 out of 35 metabolites were significantly accumulated and depleted, respectively, under elevated CO_2_ in B76. In contrast, there were equivalent proportions over total metabolites shared between up- and down-regulation by elevated CO_2_. Regarding the SHS effects, 19 and 17 out of 35 metabolites were down regulated in stressed leaves of B76 and B106, respectively, when compared with unstressed leaves (after SHS at 0 h).

**Figure 4:**
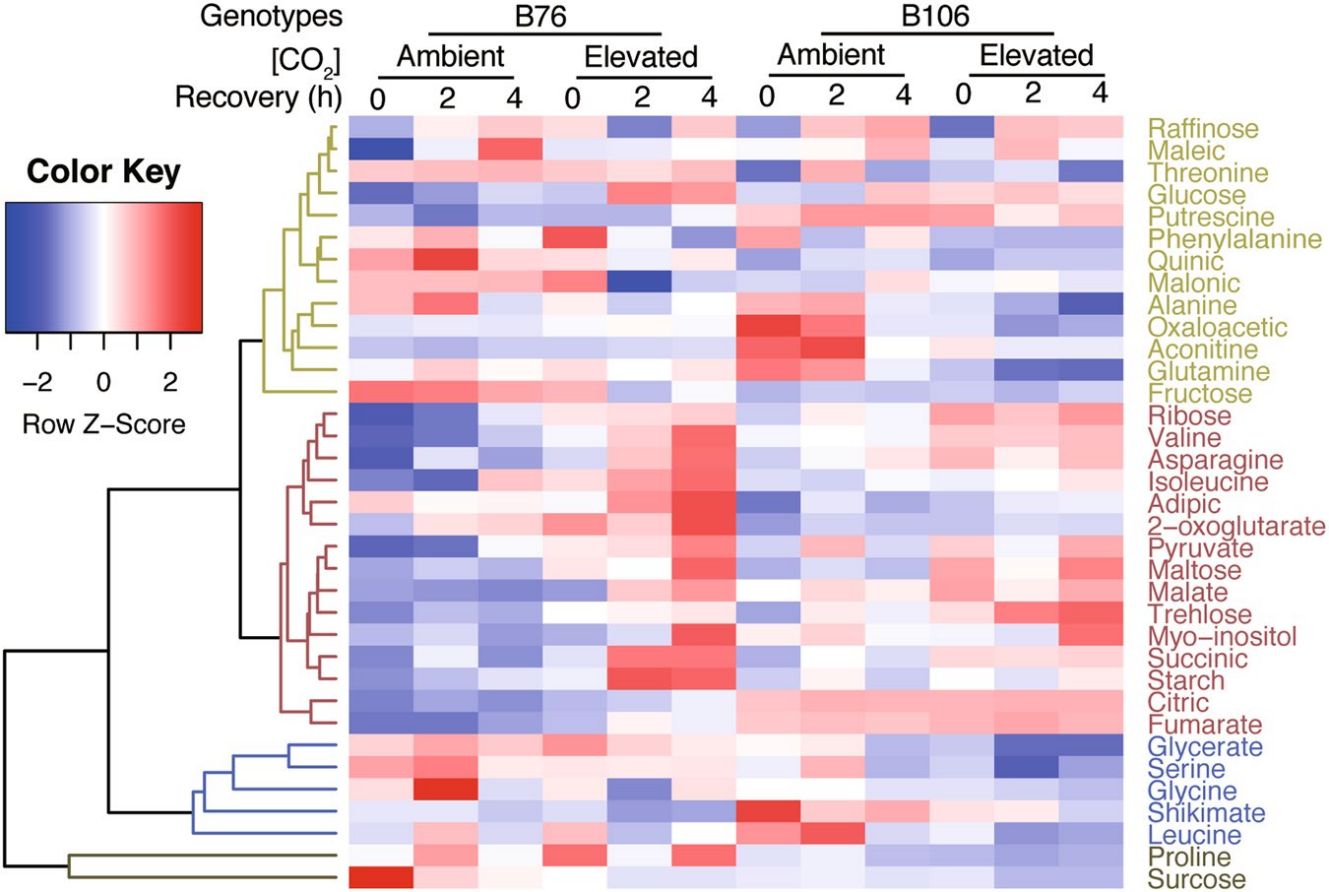
Recovery profiling following sudden heat shock (SHS) regarding to metabolites in two maize genotypes grown under ambient or elevated CO_2_. The gradient color from blue to red represent the ratio of contents of metabolites in SHS leaves over nonheated leaves for each maize genotype and CO_2_ condition. Four clusters according to the pattern along with recovery profiling of each metabolite were depicted in different colors.

In both maize genotypes, the recovery patterns of metabolites from SHS and CO_2_ treatments were ambiguous (Figs 4; S2–S4). Four clusters were characterized across CO_2_ and genotypes. In particular, cluster II represents the contents of metabolite in heat stressed leaves versus those in unstressed leaves, and the contents accumulates following recovery from SHS across CO_2_ treatments and maize genotypes. In this cluster, there were 14 metabolites, including ribose, valine, asparagine, isoleucine, adipic, 2-oxoglutarate, pyruvate, maltose, malate, trehlose, myo-inositol, starch, citric, fumarate. The starch metabolite was depleted due to SHS and started accumulating gradually with the progress of the recovery process, where B76 showed faster recovery than B106 irrespective of CO_2_ effects (Fig. S4). In contrast, there were only a few metabolites showing decreased contents following recovery phase mainly in cluster III and IV, which includes glycerate, serine, glycine, shikimate, leucine, proline, and sucrose (Fig. 4).

**Figure 5:**
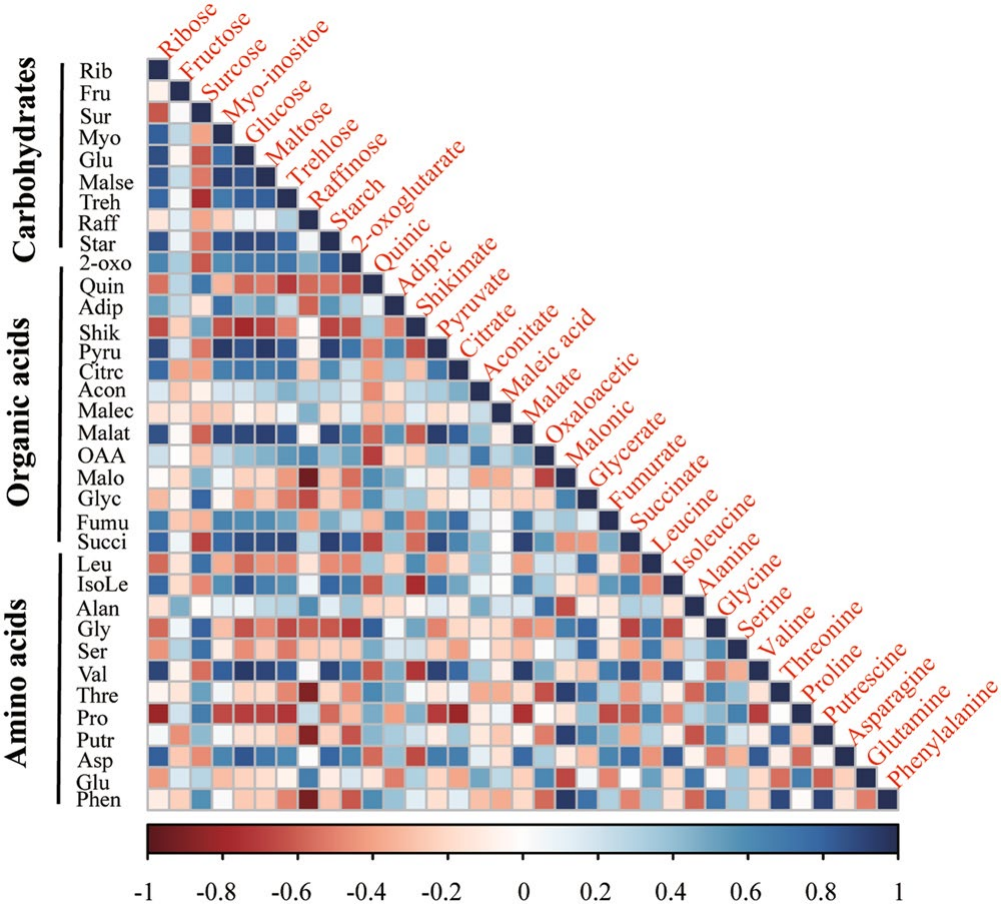
Pearson correlation between metabolites regarding carbohydrates, organic acids and amino acids across maize genotypes, recovery from sudden heat shock and CO_2_ treatments. Full name of each metabolite were presented in red color.

According to the global relatedness analysis, a robust Pearson correlation was observed among metabolites across cultivars, CO_2_ and SHS treatments (Fig. 5). Most of carbohydrates exhibited positive correlation, except sucrose and raffinose. In order to identify key metabolites indicating the response of P_N_ to SHS under two grown CO_2_ conditions across both maize genotypes, we applied a stepwise feature extraction approach, as described in materials and methods section. A linear regression model was employed. The 80% dataset was used to construct training model, while remaining 20% dataset used for test model. There were 9 key metabolites identified according to Akaike information criterion (AIC). These are: malate, valine, isoleucine, glucose, starch, sucrose, proline, glycine, and serine (Table 2). The model incorporating these metabolites explained 98% variance of P_N_ (Fig. S6; Table 2). The equation can be expressed as follows: P_N_ = Malate × 0.29 + Valine × 0.40 + Iso-leucine × 0.62 + Glucose × 0.36 + Starch × 0.37–0.25 Serine-0.14 Proline-0.82 Glycine-0.56 Sucrose (Table 2). Of these key metabolites, malate, valine, isoleucine, glucose and starch were positively correlated with recovery pattern of P_N_, while Sucrose, Proline, Glycine and Serine were opposite.

**Table 2:** Detailed parameters via feature extraction approach regarding key metabolites identified to photosynthetic rates responded to the combinations of sudden heat shock, CO_2_ and maize genotypes.

	Sq	RSS	AIC	Estimate	Std.	*t* value
(Intercept)	<none>	0.05	−99.11	**0**.**03**	0.11	0.777
Malate	0.02	0.07	−95.89	**0**.**29**	0.17	0.115
Valine	0.05	0.10	−88.24	**0**.**40**	0.13	0.013*
Isoleucine	0.08	0.14	−81.86	**0**.**62**	0.15	0.002**
Glucose	0.03	0.08	−91.80	**0**.**36**	0.15	0.035*
Starch	0.02	0.07	−95.78	**0**.**37**	0.21	0.111
Serine	0.01	0.06	−98.55	−**0**.**25**	0.21	0.269
Proline	0.04	0.09	−89.78	−**0**.**14**	0.30	0.02*
Glycine	0.08	0.13	−82.71	−**0**.**82**	0.21	0.003**
Sucrose	0.02	0.07	−93.90	−**0**.**56**	0.27	0.064

Symbols “*^,^**^,^***” represent *P* value < 0.05, 0.01 and 0.001, respectively. Note: Sum sq: Sum of squares; RSS: Residual sum of squares; AIC: Akaike information criterion; Std: standard deviation.h

### Interactive effects of CO_2_ and recovery from sudden heat shock on C_4_ photosynthetic enzymes

**Figure 6:**
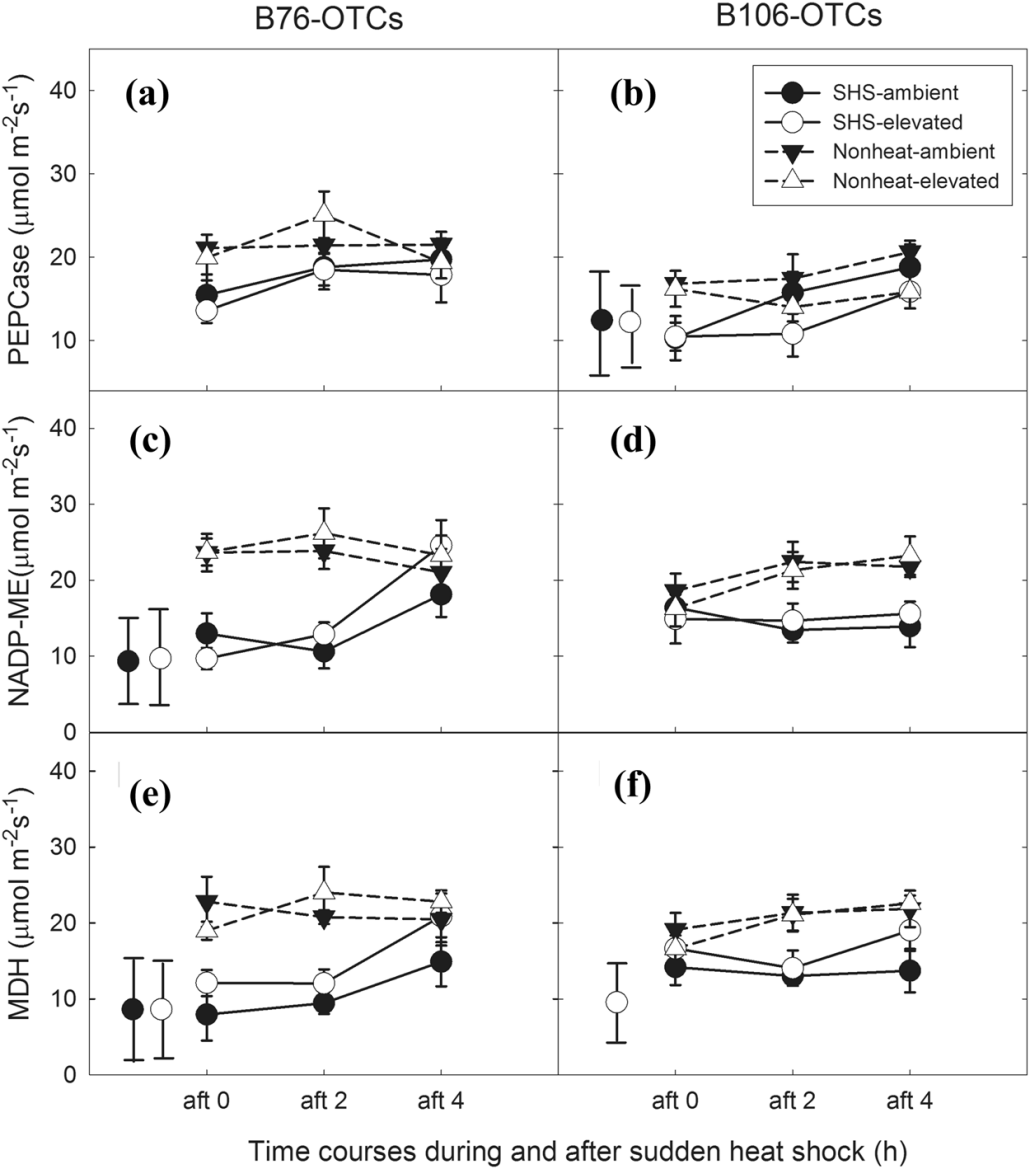
Activities of three C_4_ key photosynthetic enzymes in response to recovery from sudden heat shock (SHS) in two maize genotypes grown under two CO_2_ levels. Shapes of circle in solid line and triangle in dotted line represent SHS treated leaves and nonheated leaves, respectively, while black and white symbols mean ambient CO_2_ and elevated CO_2_, respectively. The grey area represents the period during 2 h SHS. *Vertical bars* represent at two sides of each panel represent significance scale regarding each combination of CO_2_ and SHS treatments across during and after SHS from one-way *ANOVA* analysis, (*P* < 0.05). n = 3–5.

In this study, to better understand C_4_ photosynthetic metabolism responded to SHS treatments, activities and gene expression of three key enzymes involving in C_4_ photosynthesis were determined, i.e., PEP carboxylase (PEPCase), NADP-malic enzyme (NADP-ME) and NADP-malate dehydrogenase (MDH). The activities and genes expressions of PEPCase, NADP-ME and NADP-MDH were depressed due to SHS (at 0 h) across maize genotypes (Fig. 6; Fig. S5; Table 3). PEPCase possesses better tolerance to SHS compared to the other two enzymes. B76 displayed faster recovery from SHS compared to B106 regarding the PEPCase, NADP-ME and NADP-MDH (Table 3).

**Table 3:** Comparison on the activities of three C_4_ key photosynthetic enzyme after sudden heat shock (SHS) between at 0 h (aft 0) and 4 h (aft 4) in two maize genotypes grown under ambient or elevated CO_2_.

OTCs	SHS	PEPC Activity (μmolm^−2^s^−1^)	ME Activity (μmolm^−2^s^−1^)	MDH Activity (μmolm^−2^s^−1^)
**Ambient CO2**				
B76	aft 0	15.5 ± 0.5(33.1 ± 1.6)	3.7 ± 0.4(31.6 ± 0.5)	7.9 ± 3.4(24.8 ± 3.3)
	aft 4	19.7 ± 0.5(36.5 ± 1.5)	20.6 ± 0.3(29.0 ± 0.1)	14.9 ± 1.2(26.5 ± 0.4)
	%Increase	27.1	456.8	88.6
B106	aft 0	13.3 ± 0.7(32.8 ± 0.5)	12.4 ± 1.1(24.6 ± 0.3)	17.2 ± 0.3(26.1 ± 0.2)
	aft 4	15.8 ± 0.7(36.6 ± 1.4)	13.9 ± 0.7(27.7 ± 0.3)	19.0 ± 2.7(28.9 ± 0.4)
	%Increase	18.8	12.1	10.5
**Elevated CO2**				
B76	aft 0	13.6 ± 1.5(32.0 ± 0.7)	10.9 ± 0.2(25.3 ± 0.4)	12.1 ± 1.7(29.0 ± 1.2)
	aft 4	17.9 ± 1.3(34.4 ± 0.9)	18.1 ± 1.0(26.3 ± 0.6)	20.9 ± 0.4(28.8 ± 1.5)
	%Increase	31.6	66.5	72.7
B106	aft 0	10.5 ± 1.7(32.2 ± 0.1)	9.0 ± 0.6(26.7 ± 0.8)	14.6 ± 1.7(28.6 ± 0.6)
	aft 4	12.8 ± 1.0(31.9 ± 1.0)	11.6 ± 1.6(29.2 ± 0.5)	13.7 ± 0.9(31.6 ± 1.1)
	%Increase	21.9	29.1	−6.2

Values in brackets stand for the activities of nonheated leaves. Percent increase were calculated as: [Activitiy_aft4_-Activity_aft0_/Activity_aft0_ * 100].

Elevated CO_2_ is helpful for NADP-ME and NADP-MDH, rather than PEPCase, regarding the recovery response across maize genotypes. In particular, the activities of NADP-ME in B76 under ambient CO_2_ increased by 256% measured at 4 h following SHS relative to those measured at 0 h, versus only 12% increase in B106 (Table 3). In contrast, plants grown under elevated CO_2_ showed slower recovery from SHS treatment (Table 3). B76 showed an increase by around 69% in the activities of all the three key enzymes at 4 h after ending SHS compared to those taken immediately after SHS (0 h). However, the increase in the activities of these enzymes for the same conditions was only 36% in B106 (Table 3).

### Dynamic responses of metabolites to sudden heat shock in two genotypes grown under different CO_2_ conditions

**Figure 7:**
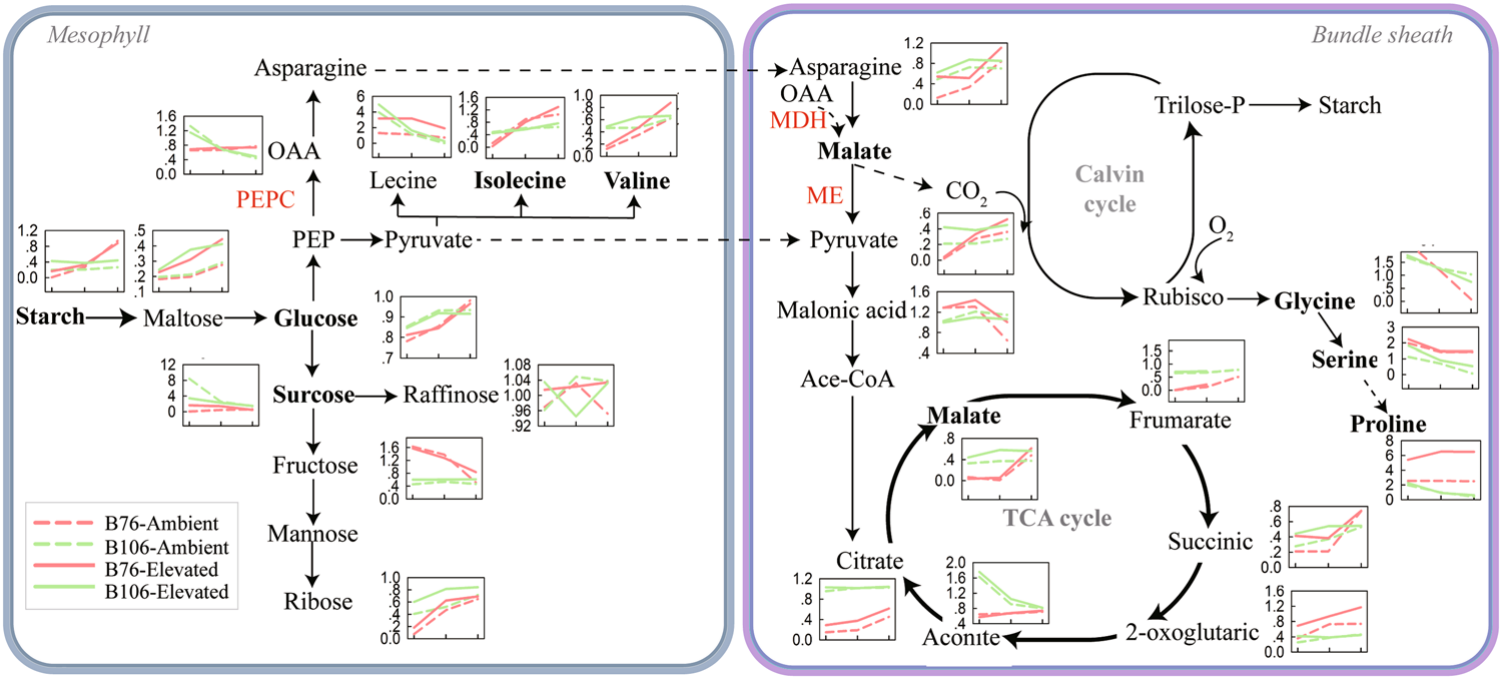
Summarized pathway of C_4_ photosynthetic metabolism in dynamic response to recovery from sudden heat shock (SHS) in two maize lines grown under different CO_2_ levels. Three C_4_ key photosynthetic enzyme were indicated in red color. In terms of each inserted panel, red and green lines represent recovery profiling of metabolites in B76 and B106, respectively, while solid and dotted line represent elevated and ambient CO_2_, respectively. The key metabolites identified by feature extraction approach that can greatly explain the photosynthetic rates in response to recovery from SHS were highlighted in bold fonts.

As shown in Fig. 7, the dynamic responses of 23 out of 35 metabolites were summarized, and there were 9 metabolites identifying for best explanation on the variance of P_N_ in response to SHS across CO_2_ and maize genotypes. These metabolites are involved in several pathways, i.e., glycolysis, malate metabolism, TCA cycle, and photorespiration. The pattern of compounds of the first three metabolic pathways were positively correlated with P_N_ response to the recovery pattern irrespective of CO_2_ and genotypes, while the last pathway (photorespiration) was negatively correlated with the P_N_ response to recovery. In particular, recovery pattern of malate and pyruvate in malate metabolism were strongly-positively correlated with recovery pattern of P_N_ following SHS. Therefore, we proposed that the malate metabolism might act important roles in recovery response for C_4_ photosynthesis from SHS.

## Discussion

Interactive effects of elevated CO_2_ and sudden heat shock (SHS) due to global climate change would dramatically and adversely affect crop productivity. Adaption of plants to such abiotic stress events mirrors the ability to maintain growth and photosynthetic efficiency from the SHS, but this adaption has been proven to be of high variability even within same species^38–40^. Photosynthetic carbon metabolism (PCM) is a key indicator to reflect acclimatory ability of plants to abiotic stress^8^. In this study, we identified 9 out of 35 metabolites as key indicators reflecting the efficiency of the photosynthetic rates (P_N_) in response to SHS via systematic biology analysis.

P_N_ is reversibly inhibited at moderately high temperature, but not at severe heat stress conditions^4,40^. Our results showed that values of P_N_ in both maize genotypes were inhibited following 2 h SHS; however the genotype B76 exhibited greater reduction in P_N_ after SHS at 0 h than genotype B106. This inhibition cannot be fully explained by stomatal limitation, due to high concentration of cellular CO_2_ (Fig. 2c,d), which was extensively observed by previous studies in other abiotic stress, such as water stress and UV-B^41–43^. In addition, CO_2_ effects on thermotolerance of B76 and B106 are distinct. Alleviation effects of elevated CO_2_ on P_N_ in B76 during and after SHS were greater than those in B106 (Fig. 2a,b). This is consistent with the fact that more metabolites were upregulated by CO_2_ in B76 rather than in B106 (Fig. 3), suggesting important functions of PCM under SHS. However, to further identify which metabolites might be key indicators reflecting the P_N_ performance following SHS requires further analysis.

Feature extraction has been extensively applied in human cancer diagnosis to identify informative metabolites or functional biomarkers^44^. In current study, we employed feature extraction approach to identify key metabolites that can greatly explain the variance of P_N_ in response to recovery from SHS incorporating into effects of CO_2_ and maize genotypes. 80% dataset was used for training dataset, and cross validation uses the remaining 20% dataset for test. After stepwise feature extraction based on Akaike information criterion (AIC), there were 9 metabolites detected, including malate, valine, isoleucine, glucose, starch, sucrose, proline, glycine, and serine (Table 2). The model incorporating these metabolites explains 98% variance of P_N_ (Fig. S6; Table 2). These metabolites are involved in various pathways, including glycolysis, malate metabolism, and photorespiratory pathway. Malic acid, an ionized form of malate, is an important intermediate-substrate of the citric acid cycle (TCA). TCA cycle provides energy resources for an optimum growth for higher plants^45^, reflecting ability of plants coping with abiotic stresses. Therefore, we will focus on discussing malate metabolism in relation with photosynthetic response to abiotic stress.

A primary site of high-temperature inhibition of Calvin cycle activity is ribulose-1,5-bisphophate carboxylase/oxygenase (Rubisco), where high temperature causes a reduction in the activation state of the enzyme^40^. However, few reports have determined whether the C_4_ photosynthetic pathway and TCA cycle were influenced by heat stress as well. In our study, we classified malate into cluster III which was dramatically inhibited by SHS at 0 h, and then appears to dramatic recovery from SHS across maize genotypes and CO_2_ treatments (Fig. 4; Fig. S2). The interactive effects between CO_2_ either with SHS or recovery on malate are significant for both genotypes (Tables S1–S2). The inhibitory effect exerted by SHS suggests that TCA is largely depressed in this case, this is in agreement with previous findings demonstrating that decreased malate levels may indicate a decline in the activity of the C_4_ photosynthetic pathway in response to drought^37^.

Malate can be synthesized by oxaloacetic acid (OAA) through a decarbonizing enzyme, NADP-malate dehydrogenase (MDH). Its activity was inhibited following immediately SHS (0 h) and recovered after SHS in B76 but its response to SHS was completely opposite in B106 (Fig. 6e,f). This is very likely ascribable to a limiting-substrate (OAA) of malate (Fig. S1), which is in line with what it has been documented previously^46^. Malate can be also converted into pyruvate through NADP-malic enzyme (NADP-ME). Not like what was observed in Arabidopsis^8^, in the current study, pyruvate amounts were decreased by SHS, leading to a reduction in the amounts of isoleucine and valine. This is probably due to the fact that enzymatic activities of NADP-ME were inhibited by SHS (Figs 6c,d and 7).

In summary, our findings suggest that B76 possesses faster recovery speed in P_N_ after removal of SHS, compared to B106. This is very likely owing to the robust metabolic flux through malate metabolic pathway in B76 via systematic biology analysis. This activated metabolic pathway stimulates TCA cycle from SHS. CO_2_ alleviating effect on P_N_ and these metabolites after SHS in B76 is possibly related to higher activities of NADP-ME and NADP-MDH in C_4_ photosynthesis. We highlighted the importance of incorporating multidisciplinary technologies to understand primary metabolism reprogramming due to environmental perturbations.

## Electronic supplementary material

Supplementary data
