# Supplementary material for: Systematic biology analysis on photosynthetic carbon metabolism of maize leaf following sudden heat shock under elevated CO2
Source: Sci Rep. 2018 May 18;8:7849. doi: 10.1038/s41598-018-26283-x (PMC5959914; doi:10.1038/s41598-018-26283-x)
Supplement: Supplementary file 1 — Supplementary data [file 41598_2018_26283_MOESM1_ESM.pdf]

Supplementary dataset

Systematic biology analysis on photosynthetic carbon metabolism of maize leaf following sudden heat shock under elevated CO_2

Mingnan Qu[1*], *Genyun Chen,*[1] *James A. Bunce*[2], *Xiaocen Zhu*[3], *Richard C. Sicher*[2]

[1]CAS Center for Excellence in Molecular Plant Sciences, Institute of Plant Physiology and Ecology, Shanghai Institutes for Biological Sciences, Chinese academy of Sciences, 200032 Shanghai, China

[2]USDA-ARS, Crop Systems and Global Change Laboratory, Beltsville, MD 20705 USA

[3]Human Phenome Institute, Fudan University, Shanghai, 200438, China.

Running title: Effects of CO_2 on C_4 metabolism recovering from heat shock

Supplementary dataset

Supplementary Table S1. Analysis of variance (*ANOVA*) for various carbohyd rates, organic acids and amino acids in response to CO_2, sudden heat shock (S HS), recovery from SHS and their interactions in B76.

B76	CO_2	SHS	Recover	CO_2*SHS	CO_2*Recover	SHSt*Recover	CO_2*SHS*Recover
Ribose	****	****	****	****	****	****	****
Fructose	**	**	****	ns	ns	****	ns
Glucose	***	****	ns	ns	ns	****	ns
My-insoitol	**	****	**	ns	ns	ns	ns
Surcose	**	****	****	ns	ns	****	ns
Maltose	****	****	****	ns	****	****	**
Trehalose	****	****	****	****	****	****	****
Raffinose	ns	ns	ns	ns	ns	ns	ns
Starch	****	****	****	****	****	****	****
2-oxoglutaric	ns	****	****	****	***	****	ns
Quinic	****	****	****	***	ns	****	*
Adipic	ns	****	*	ns	ns	*	ns
Shikimic	*	****	**	ns	ns	****	ns
Pyrumic	****	****	****	****	****	****	****
Citric	****	****	****	****	ns	****	ns
Aconitic	**	****	ns	ns	ns	ns	ns
Maleic	ns	ns	ns	ns	ns	ns	ns
Malic acid	****	****	****	****	****	****	****
Oxaloacetic	****	****	ns	ns	ns	ns	ns
Malonic	*	****	*	****	****	****	****
Alyceric	****	****	****	****	***	****	****
Fumuric	****	****	****	****	****	****	****
Succinic	****	****	****	*	ns	****	ns
Leucine	***	****	****	****	**	****	ns
Isoleucine	ns	****	****	ns	****	**	ns
Alanine	ns	ns	ns	ns	ns	ns	ns
Glycine	****	****	****	****	****	****	****

Serine	****	****	****	ns	ns	****	ns
Valine	****	****	****	****	****	****	****
Threonine	ns	ns	ns	ns	ns	ns	ns
Proline	**	****	****	****	ns	**	ns
Putrescine	ns	ns	ns	ns	ns	ns	ns
Asparagine	****	****	****	****	****	****	****
Glutamine	****	****	***	****	****	***	****
Phenylalanine	ns	*	ns	ns	ns	ns	ns

Note: Symbols “*”, “**”, “***”, “****” represent significant levels at $P<0.05$, 0.01, 0.001, or 0.0001, respectively. “ns”: not significant.

Supplementary Table S2. Analysis of variance (*ANOVA*) for various carbohydrates, organic acids and amino acids in response to CO_2, sudden heat shock (SHS), recovery from SHS and their interactions in B106.

B106	CO_2	SHS	Recover	CO_2*SHS	CO_2*Recover	SHSt*Recover	CO_2*SHS*Recover
Ribose	****	****	****	****	****	****	****
Fructose	****	****	****	***	**	ns	*
Glucose	****	****	****	ns	*	****	ns
My-insoitol	**	****	ns	ns	ns	ns	ns
Surcose	*	****	*	ns	ns	*	ns
Maltose	ns	****	****	****	****	***	****
Trehalose	****	****	****	****	****	****	****
Raffinose	ns	ns	ns	ns	ns	ns	ns
Starch	****	****	****	****	**	****	***
2-oxoglutaric	****	****	****	ns	****	ns	ns
Quinic	ns	**	*	ns	ns	ns	**
Adipic	****	****	ns	ns	*	ns	ns
Shikimic	****	****	****	ns	**	****	ns
Pyrumic	****	****	****	****	ns	ns	ns
Citric	***	ns	ns	ns	ns	ns	ns
Aconitic	**	****	****	*	****	****	ns
Maleic	ns	ns	ns	ns	ns	ns	ns
Malic acid	****	****	**	****	***	****	ns
Oxaloacetic	****	****	****	ns	****	****	**
Malonic	ns	ns	ns	ns	ns	ns	ns
Alyceric	****		****	****	****	****	****
Fumuric	**	****	ns	ns	ns	ns	ns
Succinic	****	****	*	ns	ns	**	ns
Leucine	****	****	****	ns	****	****	****
Isoleucine	****	****	*	ns	ns	*	ns
Alanine	ns	**	**	ns	ns	**	ns
Glycine	****	****	****	****	****	****	****
Serine	ns	****	****	****	***	****	****

Valine	****	****	****	****	***	****	****
Threonine	ns	ns	ns	ns	ns	ns	ns
Proline	***	****	****	ns	**	****	ns
Putrescine	ns	*	ns	ns	ns	ns	ns
Asparagine	****	****	****	****	ns	****	ns
Glutamine	****	**	****	****	****	****	****
Phenylalanine	ns	ns	ns	ns	ns	ns	ns

Note: Symbols "*", "**", "***", "****" represent significant levels at $P<0.05$, 0.01, 0.001, or 0.0001, respectively. "ns": not significant.

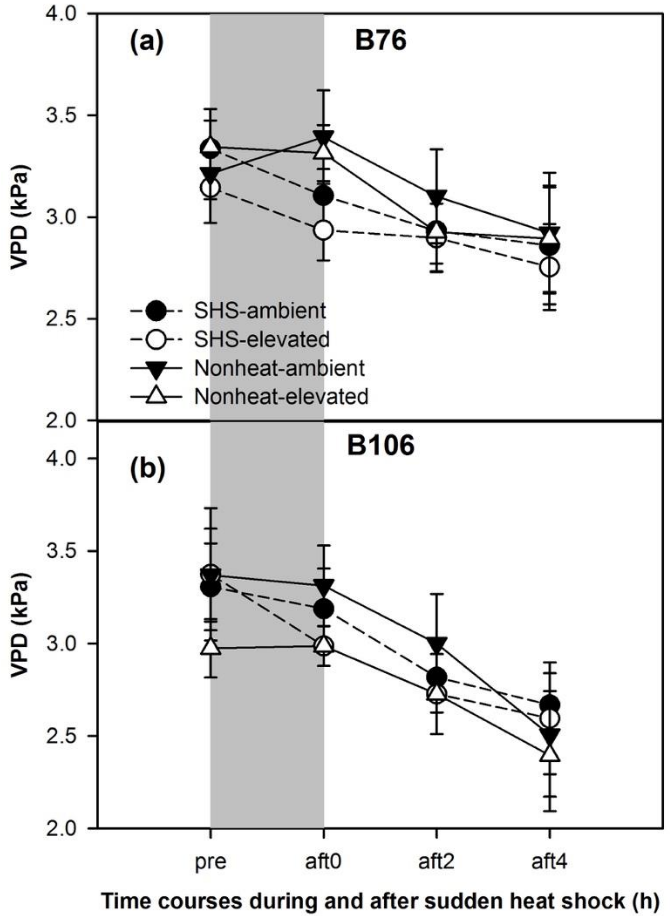

Supplementary Fig. S1. Sudden heat shock (SHS) induced decrease and recovery of vapor pressure deficit (VPD) in two maize genotypes grown under elevated CO_2 conditions. Shapes of Shapes of circle in solid line and triangle in dotted line represent SHS treated leaves and nonheated leaves, respectively, while black and white symbols mean ambient CO_2 and elevated CO_2, respectively. The grey area represents the period during 2h SHS. Vertical bars represent at two sides of each panel represent significance scale regarding each combination of CO_2 and SHS treatments across during and after SHS from one-way ANOVA analysis, ($P<0.05$). n=5

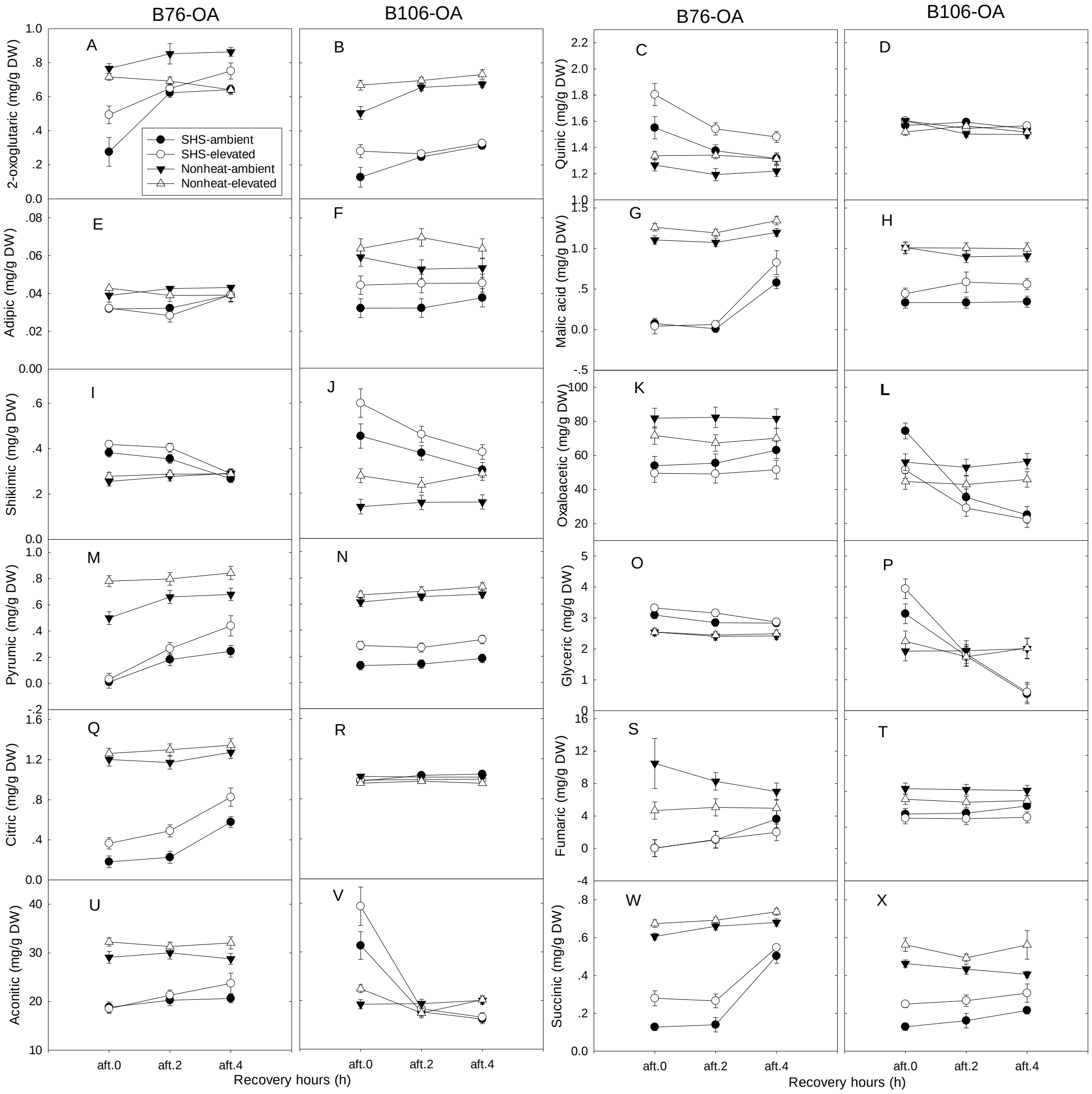

Supplementary Fig. S2. Recovery profiling of organic acids (OA) after sudden heat shock (SHS) at different hours in two maize genotypes grown under different CO_2. SHS-ambient and SHS-elevated represent response of metabolites in SHS leaves grown under ambient and elevated CO_2, respectively. Nonheat-ambient and Nonheat-elevated represent response of metabolites in nonheated leaves grown under ambient and elevated CO_2, respectively

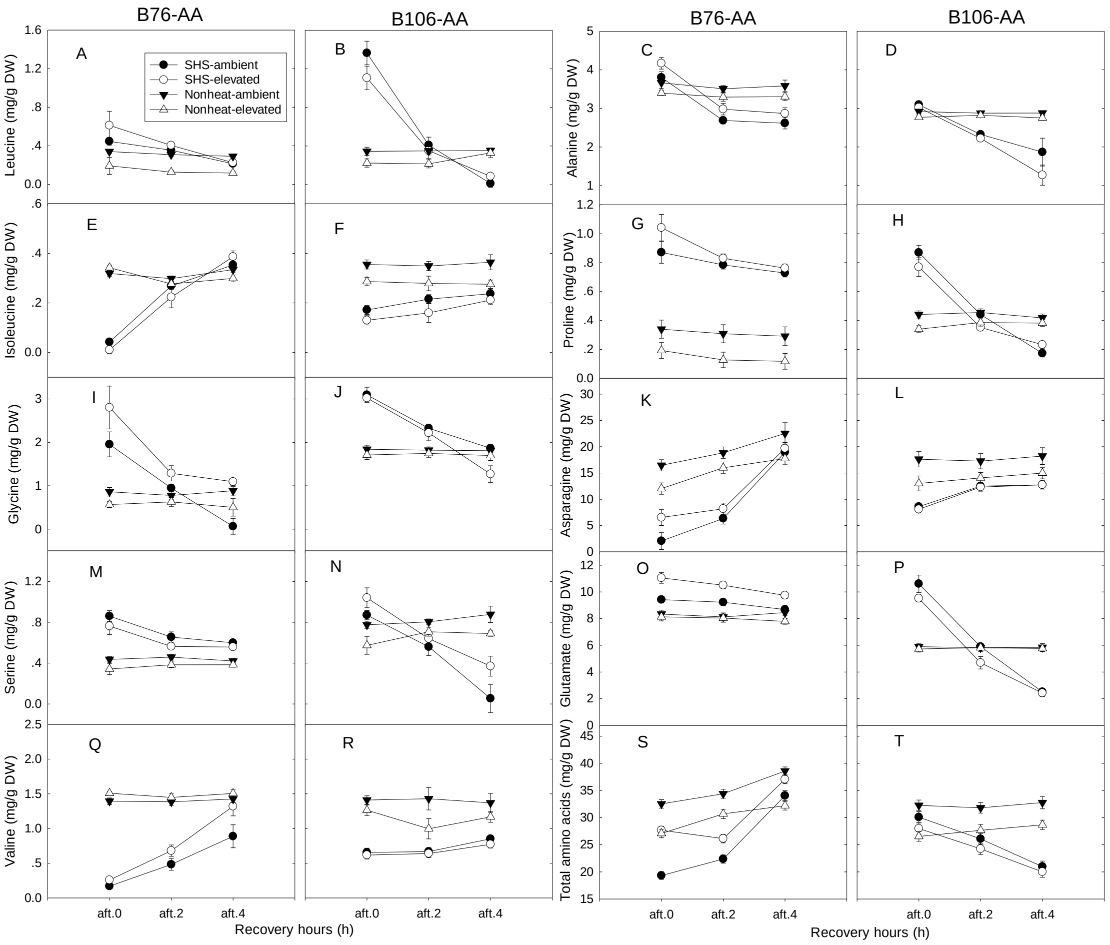

Supplementary Fig. S3. Recovery profiling of amino acids (AA) after sudden heat shock (SHS) at different hours in two maize genotypes grown under different CO_2. SHS-ambient and SHS-elevated represent response of metabolites in SHS leaves grown under ambient and elevated CO_2, respectively. Nonheat-ambient and Nonheat-elevated represent response of metabolites in nonheated leaves grown under ambient and elevated CO_2, respectively.

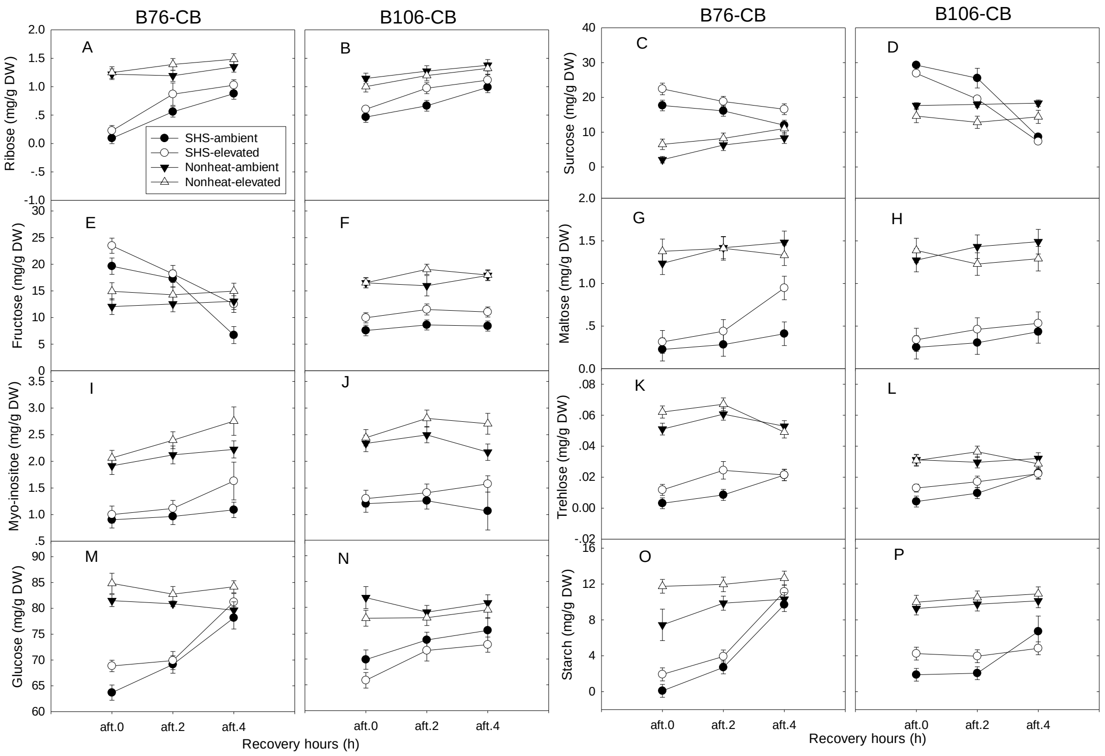

Supplementary Fig. S4. Recovery profiling of carbohydrates (CB) after sudden heat shock (SHS) at different hours in two maize genotypes grown under different CO_2. SHS-ambient and SHS-elevated represent response of metabolites in SHS leaves grown under ambient and elevated CO_2, respectively. Nonheat-ambient and Nonheat-elevated represent response of metabolites in nonheated leaves grown under ambient and elevated CO_2, respectively.

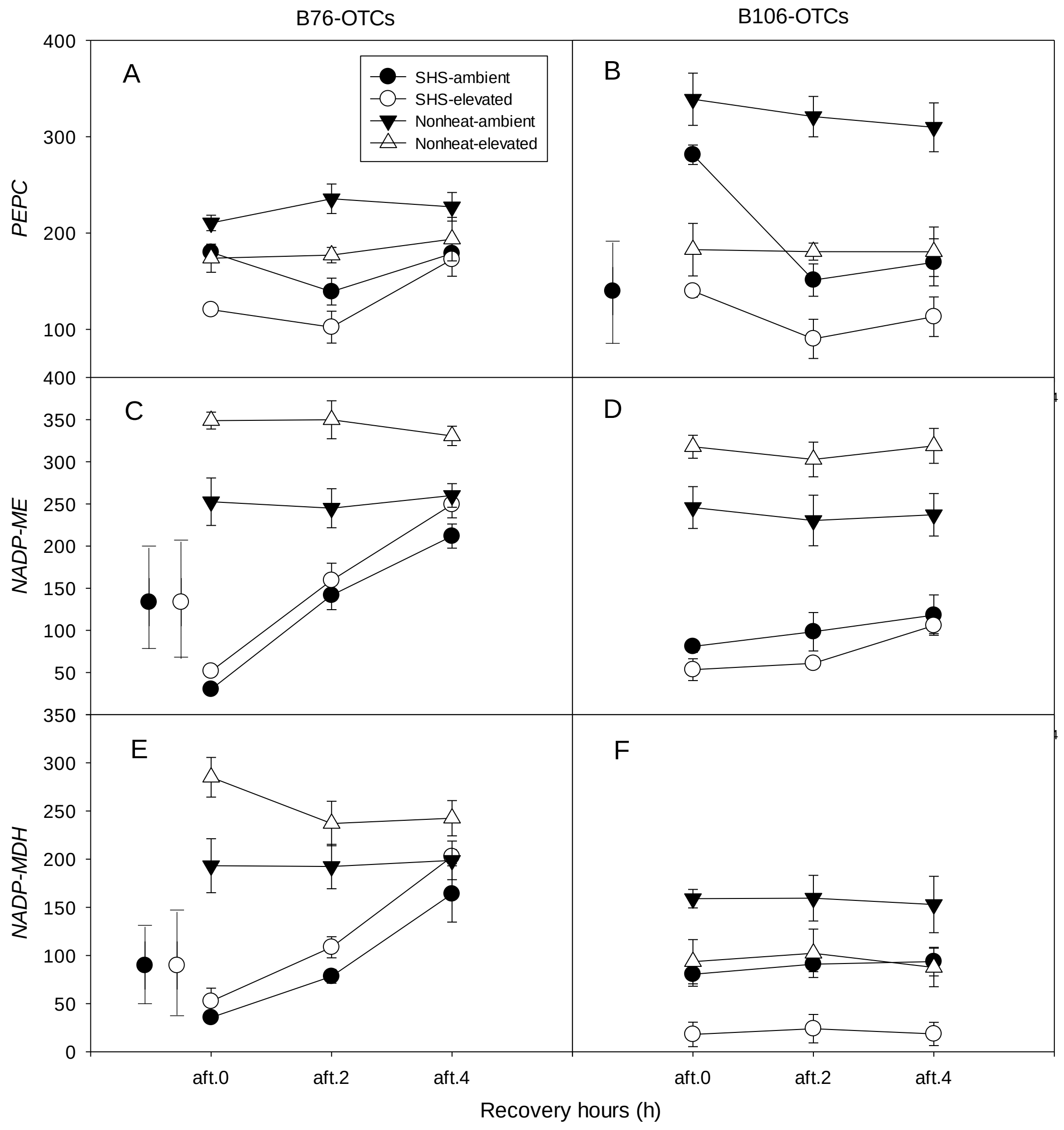

Supplementary Fig S5. Gene expression of three C_4 decarboxylating enzymes in response to recovery from sudden heat shock (SHS) in two maize genotypes grown under two CO_2 levels. Shapes of circle and triangle represent SHS treated leaves and nonheated leaves, respectively, while black and white symbols mean ambient CO_2 and elevated CO_2, respectively. The grey area represents the period during 2h SHS. *Vertical bars* represent at two sides of each panel represent significance scale regarding each combination of CO_2 and SHS treatments across during and after SHS from one-way *ANOVA* analysis, (P<0.05). n=3-5.

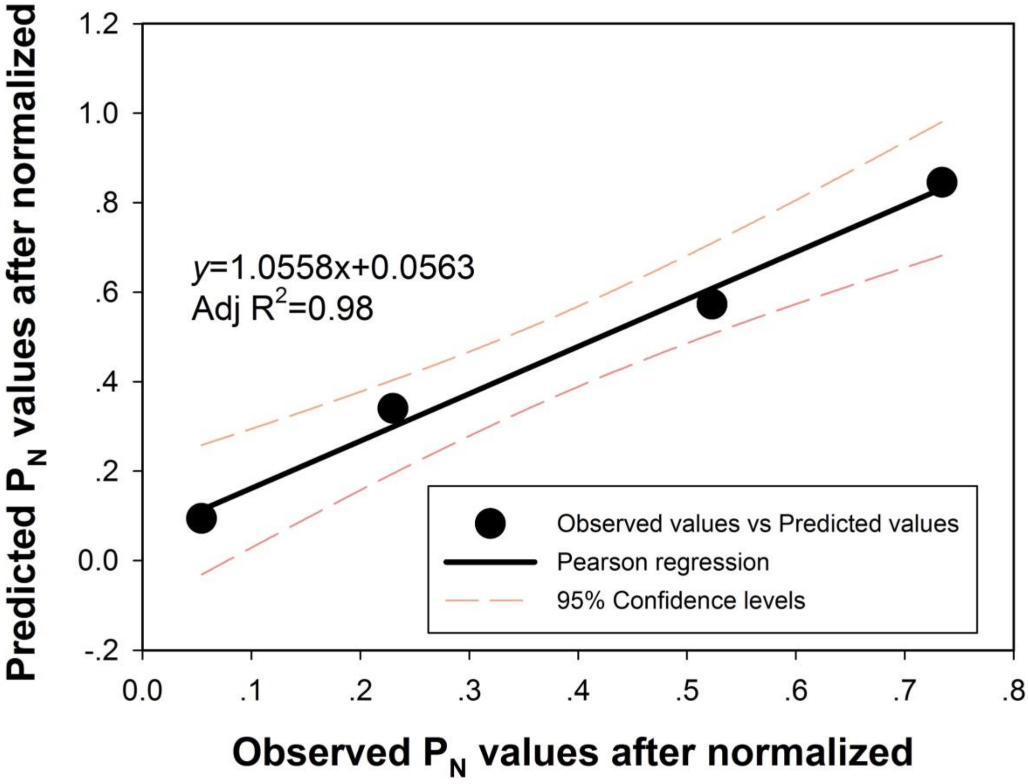

Supplementary Fig. S6. Prediction of photosynthetic rates (P_N) from test dataset using linear regression model. The values of P_N was normalized between 0~1.
